# Relationship between peripheral ischemic microvascular reserve, persistent hyperlactatemia, and its temporal dynamics in sepsis: a *post hoc* study

**DOI:** 10.5935/2965-2774.20230348-en

**Published:** 2023

**Authors:** Ana Carolina de Miranda, Fernanda do Carmo De Stefani, Bruna Cassia Dal Vesco, Hipólito Carraro Júnior, Jamil Assreuy, Luis Gustavo Morello, Igor Alexandre Cortês de Menezes

**Affiliations:** 1 Department of Internal Medicine, Hospital de Clínicas, Universidade Federal do Paraná - Curitiba (PR), Brazil; 2 Intensive Care Unit, Hospital de Clínicas, Universidade Federal do Paraná - Curitiba (PR), Brazil; 3 Department of Pharmacology, Universidade Federal de Santa Catarina - Florianópolis (SC), Brazil; 4 Instituto Carlos Chagas, Fundação Oswaldo Cruz - Curitiba (PR), Brazil

**Keywords:** Sepsis, Microcirculation, Ischemia, Hyperemia, Perfusion index, Lactate, Prognosis

## Abstract

**Objective:**

To measure the prognostic value of peripheral ischemic microvascular reserve in the context of persistent sepsis-induced hyperlactatemia and measure its influence on the temporal dynamics of lactate and the strength of association between these variables.

**Methods:**

This *post hoc* analysis of the peripheral perfusion index/postocclusive reactive hyperemia trial, an observational cohort study that enrolled patients with sepsis who persisted with lactate levels ≥ 2mmol/L after fluid resuscitation (with or without shock). Peripheral ischemic microvascular reserve was evaluated using the association of the peripheral perfusion index and postocclusive reactive hyperemia techniques. The cutoff point of ∆ peripheral perfusion index peak values (%) defined the groups with low (≤ 62%) and high peripheral ischemic microvascular reserve (> 62%).

**Results:**

A total of 108 consecutive patients with persistent sepsis-induced hyperlactatemia were studied. The high peripheral ischemic microvascular reserve group showed higher 28-day mortality than the low peripheral ischemic microvascular reserve group (p < 0.01). The temporal dynamics of lactate within the first 48 hours showed a rapid decrease in lactate levels in the low peripheral ischemic microvascular reserve group (p < 0.01). However, this result was not reproduced in the linear mixed effects model. A weak correlation between peripheral ischemic microvascular reserve (%) and lactate level (mmol/L) was observed within the first 24 hours (r = 0.23; p < 0.05).

**Conclusion:**

The prognostic value of high peripheral ischemic microvascular reserve was confirmed in the context of persistent sepsis-induced hyperlactatemia. Although there was a weak positive correlation between peripheral ischemic microvascular reserve value and lactate level within the first 24 hours of sepsis diagnosis, the low peripheral ischemic microvascular reserve group appeared to have a faster decrease in lactate over the 48 hours of follow-up.

## INTRODUCTION

Sepsis is a generalized and dysregulated immune-metabolic host response induced by an infection that culminates in organ dysfunction.^(1)^ Despite scientific advances in its pathophysiological understanding and management, this syndrome is a global problem that affects millions of patients annually and is one of the leading causes of death worldwide.^(2)^

The current understanding of sepsis holds that microcirculatory disorders such as vascular hyporesponsiveness and endothelial dysfunction are some of the mechanisms associated with the origin and progression of organ dysfunction.^(3)^ Recent evidence has shown the safety value of noninvasive bedside parameters such as the peripheral perfusion index (PPI) and postocclusive reactive hyperemia test (PORH) to evaluate microvascular reactivity through blood flow changes in response to transient flow-dependent tissue hypoxia, which may represent a “reserve” or “recruitability” of vessel control,^(4,5)^ called peripheral ischemic microvascular reserve (PIMR). A paradoxical association between higher PIMR values and poor prognosis was observed in patients with septic shock.^(4)^ This evidence is curious because it opposes the postischemic responses in tissues of patients with sepsis, such as muscle or conductance arteries.^(6)^ In addition, the pathways underlying this contradictory finding involving the high PIMR and its potential prognostic role remain unclear.

In this context, the changes in lactate levels over time in sepsis could be a possible explanation that deserves special attention. As an accepted marker of tissue hypoperfusion throughout this critical care environment, the measurement of lactate levels has been a valuable tool for dysfunctional organ recognition,^(7)^ analysis of therapeutic response,^(8)^ and making a prognosis.^(9)^ Moreover, it is well known that factors other than anaerobic metabolism can contribute to persistent sepsis-induced hyperlactatemia (PSH) after resuscitation, such as increased glycolysis, altered clearance, impaired oxygen extraction, adrenergic stimulation, or mitochondrial dysfunction.^(10)^ In this sense, it is hypothesized that persistent hyperlactatemia and its known association with worse outcomes^(9,11)^ correspond to a possible confounding variable in the association between high PIMR and higher mortality rates in sepsis. However, previous findings strongly suggest that PIMR increases the prognostic value of arterial lactate in the first 24 hours of septic shock after fluid resuscitation.^(4)^ Moreover, it is relevant to question whether the PIMR values have any influence on lactate variations over time. To answer these questions, the present observational study was designed to explore the prognostic value of PIMR in the context of persistent sepsis-induced hyperlactatemia. The two main objectives were to measure the influence of PIMR values on the temporal dynamics of lactate and measure the strength of the association between PIMR value and lactate level.

## METHODS

### Study design, setting and participants

This study was a predefined *post hoc* analysis of the PPI/PORH trial, an observational cohort study.^(12)^ The study was performed in four Brazilian intensive care units (ICUs) between November 2020 and May 2022. All survivors’ participants or their legal representatives provided written informed consent, except in the case of the patient’s death, in which case the written informed consent was waived. The Human Research Ethics Committee of the *Hospital de Clínicas, Universidade Federal do Paraná* (UFPR) approved the investigation (protocol: 3.913.982/2020) .

Consecutive adult patients (≥ 18 of age) with persistent sepsis-induced hyperlactatemia after hemodynamic resuscitation in the ICU within the first 24 hours of diagnosis were eligible for the study. The exclusion criteria applied for this study, to minimize potential confounding factors or risks of possible hemorrhagic and ischemic complications of the procedures, were pregnancy, severe hepatopathy (Child‒Pugh class C), severe coagulopathy (platelets < 20,000/mm3, international normalized ratio - RNI > 2.0, or activated partial thromboplastin time - aPTT > 70s), severe active bleeding, infective endocarditis, inaccessible perfusion assessment (severe hypothermia, Raynaud’s syndrome, or peripheral arterial occlusive disease), and refusal to participate in the study.

### Clinical definitions

#### Sepsis

According to the current sepsis guidelines, this syndrome is characterized as an infection associated with an acute alteration in the Sequential Organ Failure Assessment (SOFA) score of two points or more.^(1)^

#### Septic shock

This is a subset of sepsis wherein, despite adequate hemodynamic resuscitation with intravenous crystalloid fluid (≥ 30mL/kg),^(7)^ patients stay hypotensive (mean arterial blood pressure - MAP < 65mmHg) with signs of tissue hypoperfusion (elevated serum lactate concentration ≥ 2mmol/L).^(1)^

#### Persistent hyperlactatemia

After hemodynamic resuscitation with intravenous crystalloid fluid (≥ 30mL/kg), arterial lactate levels ≥ 2.0mmol/L characterize persistent hyperlactatemia.^(13)^

### Study protocol

All eligible patients were treated following a local standard protocol adapted from the Surviving Sepsis Campaign (SSC) guidelines.^(7)^ Treatment was initiated as soon as sepsis was diagnosed. First, if there was a high likelihood of sepsis, antimicrobials were administered within the first hour after finding suspicious focal cultures from drawn blood and specific sites according to the medical history. Second, patients with signs of hypoperfusion or septic shock received 30mL/kg of crystalloid fluid within the first 3 hours of sepsis diagnosis. This hemodynamic resuscitation was continued according to the criteria of the physician, if it was clinically indicated, until a lack of response to a passive leg raise (the cutoff value to assess fluid responsiveness was an increase in cardiac output of 10%)^(14)^ or a lack of variation in inferior vena cava diameter with breathing (the cutoff was 18% in mechanically ventilated patients and up to 42% in non-mechanically ventilated patients),^(15)^ which was estimated using a Samsung Medison Ultrasound instrument. If MAP remained < 65mmHg, norepinephrine was used to normalize this macrohemodynamic parameter. In refractory cases (noradrenaline dose > 0.5µg/kg/h), vasopressin was the drug of choice for association with noradrenaline. Hemodynamic goals consisted of a combination of criteria, such as MAP ≥ 65mmHg, diuresis > 0.5mL/kg/h, and central venous oxygen saturation (ScvO_2_) > 70%.

All patients were followed up for 28 days after sepsis diagnosis or discharge from the hospital.

### Measurements

The assessment of patients occurred within 24 hours after fluid resuscitation in patients with sepsis diagnosis. The information collected included demographic characteristics, medical history, infection source and comorbidities, Acute Physiology and Chronic Health Evolution II (APACHE II) score, and SOFA score. In addition, all hemodynamic parameters (if available), lactate levels, and peripheral variables were measured twice after fluid resuscitation: between 6 and 24 hours and between 24 and 48 hours after sepsis diagnosis. The subclavian or internal jugular vein was chosen as the location of the central venous line for ScvO_2_ and partial pressure of carbon dioxide (PCO_2_) measurements.

### Temporal dynamics of lactate

The temporal dynamics of lactate over the first 48 hours of follow-up (∆ lactate 48 hours) was established from the percentage change in lactate levels between the first (after fluid resuscitation but within 24 hours of sepsis diagnosis) and second measurements (between 24 and 48 hours of sepsis diagnosis). ∆ Lactate 48 hours was calculated using the following formula:


$$\Delta \text{ Lactate}48h=\frac{2\text{ nd lactate measurement}-1\text{ st lactate measurement}}{1\text{ st lactate measurement}}\times 100\%$$

### Assessment of peripheral ischemic microvascular reserve

This was evaluated using the association between PPI and PORH method.

Peripheral perfusion index is a parameter derived from the photoelectric plethysmography signal of a pulse oximeter, established by the light-reaching ratio between the pulsatile (arterial) and nonpulsatile components, providing a noninvasive indicator of peripheral vasomotor tone.^(16)^ It was measured after fluid resuscitation by placing a pulse oximeter probe (Masimo Radical, Masimo-Corp, Calif or MINDRAY, Shenzen, China) on the index finger. After signal stabilization, PPI was registered every 30 seconds for 5 minutes, and the average values determined the PPI basal value. Then the PORH test was conducted.

The postocclusive reactive hyperemia test is characterized by a brief arterial occlusion followed by marked vasodilation associated with a temporary increase in blood flow to the postischemic tissue.^(17)^ This test was performed after the first PPI measurement. First, a sphygmomanometer cuff was rapidly inflated around the homolateral arm to 50mmHg above the systolic pressure to occlude the arterial flow for 3 minutes. After cuff deflation, the changes in blood flow (reactive hyperemia) were measured by recording the PPI value for 5 minutes, and the highest value corresponded to the PPI peak value. Finally, the peripheral ischemic microvascular reserve was established as ∆ PPI peak (%), which was calculated using the following formula:


$$\Delta \text{ PPI peak}=\frac{\text{ PPI peak}-\text{ PPI basal}}{\text{ PPI basal}}\times 100\%$$

After selection, patients with PSH were divided into two groups of peripheral ischemic microvascular reserve using ∆ PPI peak as the cutoff point: PPI peak values below 62% (low PIMR) and above 62% (high PIMR). The cutoff point established in this study was based on the ROC curve from a previous study of septic shock patients.^(4)^

Four trained physicians conducted a clinical assessment of peripheral perfusion. This was performed in the supine decubitus position in the upper limb without an intra-arterial catheter for MAP measurement. The ambient bedside temperature in the ICU was 22ºC.

### Outcomes

The primary outcomes were the temporal dynamics of lactate levels over the first 48 hours of follow-up in the high versus low PIMR group; the correlation between PIMR values and lactate level (mmol/L) on the first and second day of sepsis diagnosis; and the correlation between the PIMR value at 24 hours (%) and ∆ lactate 48 hours (%). The secondary outcomes included the 28-day in-hospital comparison of mortality between high and low PIMR groups in the context of PSH.

### Analytical approach

All data in the present study were analyzed using IBM Statistical Package for the Social Sciences (SPSS) and GraphPad Prism 6. First, the normality of each variable was tested using the Shapiro‒Wilk test. Then, parametric data, presented as mean ± standard deviation, were compared using Student’s t test. Nonparametric data are described as medians and interquartile ranges and were compared using the Mann‒Whitney U test. Categorical data are described as frequencies and percentages and were compared using Fisher’s exact or the chi-square test (depending on the number of variables). To compare the temporal dynamics of lactate levels over the first 48 hours of follow-up between the high and low PIMR groups, the Mann‒Whitney U test (intergroup analyses) and the Wilcoxon test (intragroup analyses) were performed to evaluate the changes in lactate levels over time among patients with PSH. In addition, the Bonferroni test was used for multiple comparisons. Furthermore, an additional analysis of the temporal dynamics of lactate levels over time was performed. For this analysis, the groups defined by low or high PIMR on two consecutive days (Days 1 and 2) were compared, and the interaction between group and day was evaluated. For this purpose, a linear mixed effects model with random intercept and slope was adjusted. Group was considered a fixed effect, and patient was considered a random effect. For this analysis, the lactose data were subjected to a logarithmic transformation. Finally, Spearman’s correlation coefficient was calculated between continuous variables. All reported p values are two-sided; p < 0.05 was statistically significant.

Cohen’s Kappa test established the agreement of the study devices.

As this was a post hoc study, the sample consisted of all patients from the original study who met the inclusion criteria.

This study followed the STROBE guidelines for reporting results.

## RESULTS

### Baseline characteristics of the study population

**Figure 1:**
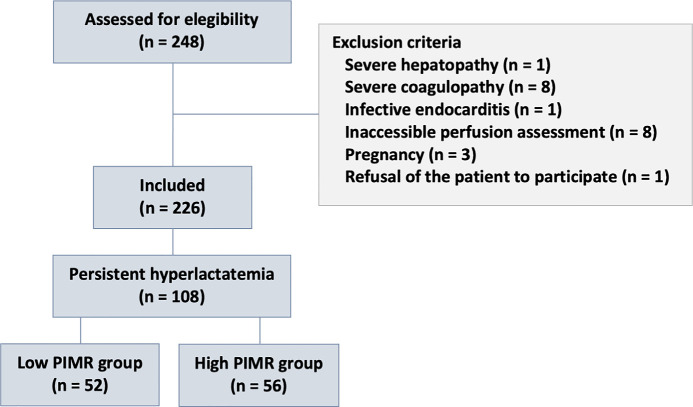
Peripheral perfusion index/postocclusive reactive hyperemia test patients with persistent sepsis-induced hyperlactatemia included in the analysis.

During the study period, 118 of the 226 patients with PSH and were considered eligible for inclusion after fluid resuscitation and underwent subsequent peripheral microvascular reserve assessment (Figure 1). The demographics and characteristics of the study population are presented in table 1. Collectively, these data describe a heterogeneous critically ill population, which is a typical finding given sepsis. Both groups presented stable hemodynamic parameters at the assessment time. The high PIMR group had a higher portion of patients with a previous history of cerebrovascular disease and abnormal peripheral perfusion parameters after fluid resuscitation than the low PIMR group. There were no significant differences between groups (high and low PIMR) in other clinical data (age, sex, other comorbidities), infection source, confirmed culture, clinical severity scores (APACHE II, SOFA), ICU admission biomarkers, lactate levels, or vasoactive drug use.

**Table 1:** Baseline characteristics of patients with persistent sepsis-induced hyperlactatemia

Parameters	All patients n = 108	Low PIMR group n = 52	High PIMR group n = 56	p value
Age (years)	62 ± 16	60 ± 16	63 ± 15	0.218
Sex				0.255
Men	56 (51.9)	30 (57.7)	26 (46.4)	
Women	52 (48.1)	22 (42.3)	30 (53.6)	
Comorbidities				
Diabetes mellitus	38 (35.2)	18 (34.6)	20 (35.7)	1.000
Hypertension	60 (55.6)	28 (53.8)	32 (57.1)	0.847
Chronic kidney disease	7 (6.5)	3 (5.8)	4 (7.1)	1.000
Heart failure	14 (13)	6 (11.5)	8 (14.3)	0.778
Liver failure	7 (6.5)	4 (7.7)	3 (5.4)	0.709
Cerebrovascular disease	10 (9.3)	1 (1.9)	9 (16.1)	0.017^*^
Chronic pulmonary disease	14 (13)	7 (13.5)	7 (12.5)	1.000
Cancer	15 (13.9)	6 (11.5)	9 (16.1)	0.584
Immunosuppression	23 (21.3)	8 (15.4)	15 (26.8)	0.166
Source of infection				
Respiratory	48 (44.4)	22 (42.3)	26 (46.4)	0.702
Abdominal	29 (26.9)	14 (26.9)	15 (26.8)	1.000
Urinary	14 (13)	9 (17.3)	5 (8.9)	0.408
Others	17 (15.7)	7 (13.5)	10 (17.9)	0.790
Any microorganism in cultures	71 (65.7)	37 (71.2)	34 (60.7)	0.312
Confirmed bloodstream infection	31 (28.7)	16 (30.8)	15 (26.8)	0.676
Scores and biomarkers at ICU admission				
SOFA†	9 ± 4	9 ± 4	10 ± 4	0.362
APACHE II‡	24 ± 9	23 ± 1	25 ± 8	0.305
CRP (mg/dL)	16 (10.6 - 20.8)	17 (8 - 21.1)	16 (12 - 20)	0.951
Procalcitonin (ng/mL)	81/3 (0.6 - 11.5)	37/3.3 (0.3 - 12.7)	44/2.2 (0.9 - 10.2)	0.780
Hemodynamic data after resuscitation				
MAP (mmHg)	82 (74 - 92)	81 (71 - 88)	85 (75 - 92)	0.387
Heart rate (/minute)	98 ± 22	98 ± 23	98 ± 20	0.963
ScvO_2_ (%)	40/73 ± 10	18/73 ± 11	22/73 ± 9	0.961
Pv-aCO_2_ (mmHg)	40/7.1 (6.9)	18/6.9 (6.6)	22/7.3 (7.3)	0.851
Urine Output (mL/kg/h)	105/0.5 (0.2 - 0.9)	50/0.5 (0.4 - 0.9)	55/0.5 (0.2 - 0.9)	0.457
Lactate (mmol/L), 1rt measurement	2.7 (2.3 - 3.8)	2.6 (2.2 - 3.6)	3.1 (2.5 - 3.8)	0.058
Lactate (mmol/L), 2^nd^ measurement	2.5 (1.9 - 3.4)	2.2 (1.7 - 3.0)	2.7 (2.0 - 3.9)	0.053
Vasoactive drugs use	70 (64.8)	33 (63.5)	37 (66.1)	0.841
Noradrenaline dose (µg/kg/min)	69/0.3 (0.2 - 0.5)	32/0.2 (0.1 - 0.5)	37/0.3 (0.2 - 0.6)	0.064
Vasopressin use	21 (19.4)	8 (15.4)	13 (23.2)	0.340
Abnormal peripheral perfusion				
Prolonged CRT (> 3s)	45 (41.7)	14 (26.9)	31 (55.4)	0.003§
Altered PPI (< 1.4)	51 (47.2)	10 (19.2)	41 (73.2)	< 0.001§
Mortality	57 (52.8)	20 (38.5)	37 (66.1)	0.007§

PIMR - peripheral ischemic microvascular reserve; ICU - intensive care unit; SOFA - Sequential Organ Failure Assessment; APACHE II - Acute Physiology and Chronic Health System II; CRP - C-reactive protein; MAP - mean arterial pressure; ScvO_2_, - central venous oxygen saturation; Pv-aCO_2_ - venous to arterial carbon dioxide difference; CRT - capillary refill time; PPI - peripheral perfusion index.

* p < 0.05; † range, 0 to 24: higher scores are associated with the intensity of organ dysfunction and a higher risk of in-hospital death.^(24)^ ‡ Range, 0 to 71: higher scores are associated with the intensity of illness and a higher risk of in-hospital death.^(24)^ § p < 0.01. The results are expressed as mean (standard deviation), n (%), median (interquartile range), n/median (interquartile range) or n/mean (standard deviation).

PIMR - peripheral ischemic microvascular reserve.

The 28-day in-hospital mortality of the high PIMR group was 66.1%. (37/57), which was higher than that of the low PIMR group (38.5%, 20/57) (p < 0.01).

### Reliability among measurements obtained by two pulse oximeters

The agreement between the two pulse oximeter probes, the Masimo Radical (Masimo-Corp, CA, USA) and MINDRAY (Shenzen, China), demonstrated a moderate concordance (Cohen’s kappa = 0.76; p < 0.01).

### Temporal analyses of lactate levels between high and low peripheral ischemic microvascular reserve groups

**Figure 2:**
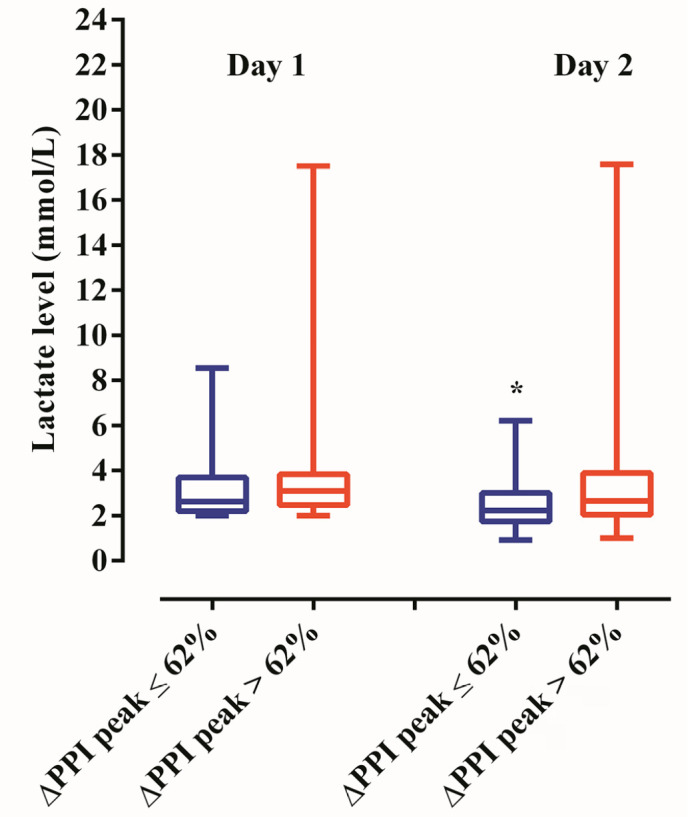
Temporal evaluation of lactate levels between high and low peripheral ischemic microvascular reserve groups in persistent sepsis-induced hyperlactatemia patients.

As shown in figure 2, we performed serial lactate assessments in patients with PSH. This analysis used the nonparametric study model including patients with complete data over the first two days after fluid resuscitation and compared the high and low PIMR groups (n = 84). There was no significant difference between the groups within 24 hours (p = 0.15) or 48 hours (p = 0.05). Moreover, in intragroup analyses, statistically relevant changes in lactate levels were observed over time in the high PIMR group (p = 0.02) and in the low PIMR group (p < 0.01). Only the low PIMR group demonstrated an effective reduction in lactate levels over time after adjusting for multiple comparisons.

Values depicted are medians and interquartile ranges. Intergroup analysis (low *versus* high peripheral ischemic microvascular reserve): Day 1 and Day 2 not significant. Intragroup analysis of lactate levels over 48 hours of follow-up: high peripheral ischemic microvascular reserve group not significant; low peripheral ischemic microvascular reserve group; * p < 0.05. Statistical tests: the Mann-Whitney U test (intergroup analyses), the Wilcoxon test (intragroup analyses), and the Bonferroni post hoc test were used for multiple comparisons.

PPI - peripheral perfusion index; ∆ PPI peak - variation of peripheral perfusion index peak.

**Figure 3:**
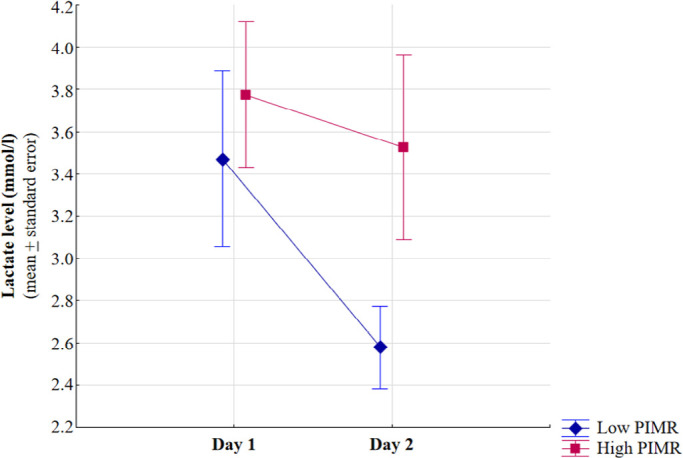
The temporal dynamics of lactate levels over the first 48 hours of follow-up between the high and low peripheral ischemic microvascular reserve groups. Group and time interaction analyses not significant. Intergroup analyses (high and low peripheral ischemic microvascular reserve) not significant. Intragroup analyses: p < 0.01. Statistical tests: linear mixed effects model.

As shown in figure 3, serial lactate assessment was performed during the first 48 hours in patients with PSH after fluid resuscitation (n = 108) with missing data (linear mixed effects model). There was no interaction between day and group (p = 0.21). There were no significant differences between groups throughout the entire evaluated period (p = 0.89). A significant alteration in lactate levels over time was observed within each group (p < 0.01).

PIMR - peripheral ischemic microvascular reserve.

**Correlation between peripheral ischemic microvascular reserve and changes in lactate levels over time**

**Figure 4:**
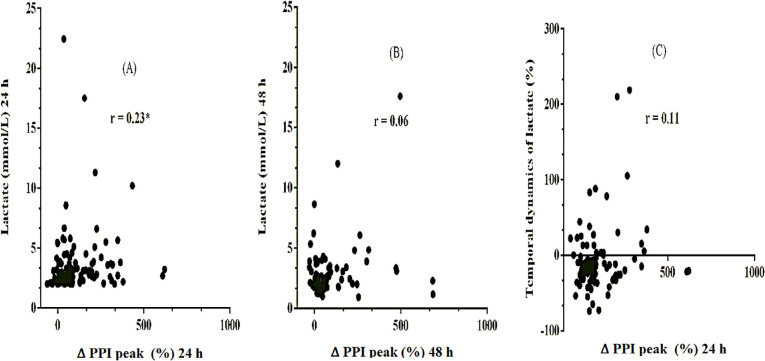
Analysis of the strength of association between the variables PIMR and lactate level and the temporal dynamics of lactate. (A) Correlation between peripheral ischemic microvascular reserve (variation of peripheral perfusion index peak %) and lactate level (mmol/L) in patients with persistent sepsis-induced hyperlactatemia after fluid resuscitation within 24 hours of sepsis diagnosis. * p < 0.05; (B) Correlation between the value of peripheral ischemic microvascular reserve (variation of peripheral perfusion index peak %) and lactate level (mmol/L) on the second day of evaluation not significant. (C) Correlation between the value of peripheral ischemic microvascular reserve (variation of peripheral perfusion index peak %) and the temporal dynamics of lactate (%) not significant.

As shown in figure 4A, there was a weak (r = 0.23) but statistically significant positive correlation between the ∆ PPI peak and lactate level (mmol/L) within the first 24 hours of the sepsis diagnosis after fluid resuscitation (p = 0.01). However, as demonstrated in figure 4B, there was no significant correlation between these variables on the second day (p = 0.55). In addition, as shown in figure 4C, there was no significant correlation (p = 0.31) between the ∆ PPI peak on the first day and the temporal dynamics of lactate (%).

PIMR - peripheral ischemic microvascular reserve; ∆ PPI peak - variation of peripheral perfusion index peak.

## DISCUSSION

Concerning the essential role of microcirculatory disorders in the pathophysiology of sepsis, particular attention has been paid to the impairments in microvascular reactivity linked to organ failure in human sepsis.^(18)^ Recently, Menezes et al. demonstrated, throughout the skin response to transient ischemia, the value of high PIMR in predicting 28-day mortality in septic shock patients.^(4)^ However, the mechanisms underlying this paradoxical finding remain unclear. Hence, the present study provides new evidence regarding the reproducibility of the previous evidence in the context of persistent sepsis-induced hyperlactatemia (with and without shock) and a possible explanation for the potential prognostic value of high PIMR based on the temporal dynamics of lactate.

Throughout the critical environment of sepsis, microcirculatory blood flow disturbances are unequivocally recognized as one of the mechanisms responsible for the induction of anaerobic metabolism of glucose and consequently explain high lactate levels.^(10)^ Therefore, some steps in sepsis management, such as early antibiotic therapy and fluid administration, are crucial for improving the prognosis of sepsis.^(7)^ However, once hemodynamic resuscitation has been established (the time of PIMR assessment chosen in this study), it is questionable whether flow-dependent tissue hypoxia remains the primary cause of persistently high lactate and why high lactate has low prognostic value. This doubt was reinforced through our results, which showed that approximately half of the sepsis population (108/226) had high lactate levels despite stable macrohemodynamic parameters during the PIMR assessments.

Thus, given the complexity of this biomarker, this investigation sought to assess whether, in addition to the metabolic disturbances currently recognized in the development of persistent hyperlactatemia, such as loss of a tissue’s ability to extract oxygen secondary to microvascular endothelial damage and tissue edema, mitochondrial and enzymatic dysfunctions, increased glycolysis exceeding metabolizing capacity, adrenergic stimulation, and reduced clearance attributed to sepsis-associated organ dysfunction,^(10)^ the PIMR values would have some influence on changes in lactate levels over time. Interestingly, through temporal dynamics of lactate analyses over the first 48 hours (after fluid resuscitation) in the groups with high and low PIMR, the present study showed that only patients with low PIMR effectively reduced their lactate levels over time. Here, one methodological aspect deserves to be highlighted. As the model chosen for the serial analysis of lactate (proof-of-concept) did not allow for missing data, a lactate dynamics curve was also constructed using a mixed effects model, which allowed the inclusion of incomplete data on both days. Using this approach, these differences in lactate dynamics between groups ceased to exist. This suggests that although the finding of low PIMR is related to lower lactate levels over time, this test does not seem to have independent predictive value in the first 24 hours. In addition, given these results, we sought to investigate the strength of the association between the variables PIMR value and lactate level over the 48 hours of follow-up. A weak positive correlation was verified between the variables within the first 24 hours, suggesting that PIMR values (after hemodynamic resuscitation with fluids within 24 hours of sepsis diagnosis) possibly interfere with lactate levels in this period. However, no significant associations were observed between lactate levels and PIMR values at 48 hours.

Collectively, these findings suggest some possible interpretations: (a) despite the known structural damage of sepsis in the microcirculation, essential functionality persists in vasoreactivity, at least in cutaneous tissue;^(4)^ and (b) if these findings occur concurrently in vital organs, we can assume that patients in the low PIMR group have microvascular recruitment optimized for local tissue flow demands, which may decrease lactate production or increase its clearance. However, considering that the persistence of hyperlactatemia in sepsis is due more to increased lactate production than clearance,^(19)^ our research cannot distinguish patients who produce less lactate from those who clear it faster. (c) The relationship of PIMR with the adrenergic effect could also contribute to the slower drop in lactate in the group with high PIMR, whereas in addition to increased glucose metabolism, overloading the system and consequently contributing to the persistent sepsis-induced hyperlactatemia,^(10)^ the levels of catecholamine (norepinephrine) correlate positively with PIMR values, as demonstrated in the study by Menezes et al.^(4)^ Therefore, the stimulation of adrenergic receptors by vasoactive mediators, used by most PSH patients (64.8%), in addition to interfering in microvascular reactivity, could also explain the variations of lactate levels in the low PIMR group. Moreover, the positive association between PIMR and adrenergic mediators^(4)^ could also contribute to the slower drop in lactate levels in the high PIMR group, indicating the persistence of sympathetic stimuli compensatory effects or autonomic dysfunction. Further investigations are needed to test this hypothesis.

Regardless of the pathophysiological interpretation of this phenomenon, these results highlight the prognostic enrichment of PIMR in patients with PSH. This information can help the clinician consider the benefit-risk ratio, whereby only therapies carrying minimal risks may be justified for patients with a higher likelihood of the outcome^(20)^ in question, mortality, aiming at more personalized treatment and better prediction of the outcome of sepsis. Thus, selecting subgroups of patients with different outcomes (high and low PIMR) and presenting similar pathophysiological characteristics (PSH) would increase the chance of response to marker-guided therapies and survival.^(20)^ Moreover, this study used a bedside test to support the early identification of a subgroup of patients with PSH whose mortality was twice as high and was indistinguishable from other clinical/hemodynamic parameters (high PIMR group). Our data add valuable information on the role of cutaneous microvascular reactivity in patients with high-risk hyperlactatemia.^(21)^ Recently, it was observed that the association of hyperlactatemia with prolonged capillary refill time exponentially increases the risk of death.^(21)^ New studies need to be carried out on these two variables. In this sense, through the PIMR value, intensivists could identify a subgroup in whom both fluid resuscitation and vasoactive drug administration should be used with greater caution (high PIMR group) and could theoretically avoid the damage associated with iatrogenic excessive fluid administration or improper administration of vasoactive drugs targeting higher MAP, known clinical worsening factors,^(22,23)^ in those lower-risk patients (low PIMR group). Additionally, these results open new perspectives for investigating the possible causes of poor prognosis in high PIMR patients, such as insufficient resuscitation, autonomic dysfunction, and mitochondrial dysfunction.

The current study had some limitations. First, it used a predefined post hoc analysis from the PPI/PORH trial. Thus, no sample size was calculated based on the mortality rates of patients with persistent hyperlactatemia and a high PIMR. For this reason, new studies are necessary to confirm our findings. Second, blood lactate measurements were not performed at the same time interval in all patients in the analysis of the temporal dynamics of lactate limits, at least some of the time in the interindividual comparative analysis. However, because all patients’ macrohemodynamics were stabilized in this period (postresuscitation), the lactate fluctuations in this interval can be considered smaller than in the initial phase of the syndrome. Third, although patients were assessed consecutively and included septic patients of various clinical severities, 2/3 of the patients had septic shock. Thus, the study findings cannot be generalized to patients without shock or in settings outside the ICU. In addition, multivariate analysis was not performed. The main study^(12)^ is being finalized to verify the value of elevated PIMR as an independent prognostic factor for mortality, which would answer these and other questions. Finally, the possible confounder variables, such as the proportion of mechanically ventilated patients and the proportion of patients with cardiac arrhythmias that were not obtained, limit the value of PIMR as a prognostic factor. However, robust evidence has shown that although the PPI parameter may suffer from the influence of these variables, it maintains its prognostic value for mortality in sepsis,^(24)^ probably because the most important predictive factor is the resulting blood flow at the microcirculatory level (regardless of the factors that alter it).

## CONCLUSION

Peripheral ischemic microvascular reserve assessment seems to be a potential tool for the early identification of a subgroup of patients with persistent hyperlactatemia who have a higher risk of death. In addition, although there was a weak positive correlation between peripheral ischemic microvascular reserve values and lactate levels within the first 24 hours after the sepsis diagnosis, the low peripheral ischemic microvascular reserve group appeared to have a faster decrease in lactate levels over the 48 hours of follow-up. Further studies are needed to check its prognostic value and elucidate its pathophysiological mechanisms.
